# Simulating the spread of selection-driven genotypes using landscape resistance models for desert bighorn sheep

**DOI:** 10.1371/journal.pone.0176960

**Published:** 2017-05-02

**Authors:** Tyler G. Creech, Clinton W. Epps, Erin L. Landguth, John D. Wehausen, Rachel S. Crowhurst, Brandon Holton, Ryan J. Monello

**Affiliations:** 1 Department of Fisheries and Wildlife, Oregon State University, Corvallis, Oregon, United States of America; 2 Computational Ecology Laboratory, Division of Biological Sciences, University of Montana, Missoula, Montana, United States of America; 3 White Mountain Research Center, University of California, Bishop, California, United States of America; 4 Grand Canyon National Park, National Park Service, Grand Canyon, Arizona, United States of America; 5 Biological Resources Division, National Park Service, Fort Collins, Colorado, United States of America; Australian National University, AUSTRALIA

## Abstract

Landscape genetic studies based on neutral genetic markers have contributed to our understanding of the influence of landscape composition and configuration on gene flow and genetic variation. However, the potential for species to adapt to changing landscapes will depend on how natural selection influences adaptive genetic variation. We demonstrate how landscape resistance models can be combined with genetic simulations incorporating natural selection to explore how the spread of adaptive variation is affected by landscape characteristics, using desert bighorn sheep (*Ovis canadensis nelsoni*) in three differing regions of the southwestern United States as an example. We conducted genetic sampling and least-cost path modeling to optimize landscape resistance models independently for each region, and then simulated the spread of an adaptive allele favored by selection across each region. Optimized landscape resistance models differed between regions with respect to landscape variables included and their relationships to resistance, but the slope of terrain and the presence of water barriers and major roads had the greatest impacts on gene flow. Genetic simulations showed that differences among landscapes strongly influenced spread of adaptive genetic variation, with faster spread (1) in landscapes with more continuously distributed habitat and (2) when a pre-existing allele (i.e., standing genetic variation) rather than a novel allele (i.e., mutation) served as the source of adaptive genetic variation. The combination of landscape resistance models and genetic simulations has broad conservation applications and can facilitate comparisons of adaptive potential within and between landscapes.

## Introduction

The field of landscape genetics has greatly enhanced our understanding of the influence of landscape composition and configuration on gene flow and genetic variation of organisms [1]. The most common product of landscape genetic studies is the landscape resistance model, which represents the cost of movement, reduction in survival, or willingness of an animal to move through the environment as a function of landscape characteristics such as cover type, topography, or degree of anthropogenic disturbance [2]. Landscape resistance models are developed using neutral genetic markers, which are ideal for investigating demographic processes such as gene flow, migration, and dispersal because neutral variation is not influenced by selective forces that can lead to incorrect inferences about these processes [3]. However, many of our most pressing questions about the effects of landscape characteristics on species and populations concern adaptive genetic variation–the ultimate driver of evolutionary potential–and understanding how landscape characteristics affect the potential for spread of adaptive variation is a pressing need in landscape genetics [4, 5]. This need will only increase as unprecedented rates of habitat modification [6] and climate change [7] force many species to adapt to novel environmental conditions. Additionally, rapid advances in next-generation sequencing technology are making it much easier to identify adaptive loci and explore genotype-environment associations (e.g., [8]).

Much of our understanding of how landscape characteristics could influence the spread of adaptive variation comes from theoretical models, such as those that explore effects of population subdivision on the rate or probability of fixation of a beneficial mutation. For instance, beneficial mutations spread at a slower rate in structured populations [9] and reach fixation faster when demes are two-dimensionally structured than one-dimensionally structured [10]. Yet, theoretical models tend to rely on simplifying assumptions about the spatial arrangement of populations and the nature of migration between them (e.g., island, stepping-stone, or lattice models of population structure) that are rarely borne out in real life. Characteristics of the intervening landscape between individuals or populations (e.g., habitat configuration, presence of dispersal barriers) are well known to limit animal movement and gene flow and ultimately affect the amount and spatial pattern of genetic differentiation [11–14]; these characteristics must be taken into account when assessing how real landscapes influence adaptive variation.

Selection strength also plays a role in determining how adaptive variation spreads across populations [15, 16]. Theoretical research suggests that an advantageous allele can spread rapidly through a subdivided population with very low gene flow if its selective advantage is strong [9]. Selection coefficients for some quantitative trait loci in animals are believed to be sufficiently high to allow for rapid spread of these traits and evolution at the species level [17].

Landscape resistance models based on neutral genetic variation can play an important role by providing realistic, empirically-supported backdrops for simulating the spread of adaptive genetic variation across landscapes. Individual-based, spatially-explicit genetic simulators now allow us to incorporate natural selection in the simulation of gene flow and demographic processes across resistant landscapes (e.g., [18, 19]). With many species widely distributed across landscapes that vary dramatically with respect to landscape characteristics, simulation-based comparisons within and among landscapes could help to identify portions of a species’ range where adaptive alleles are likely to spread quickly and facilitate in-situ adaptation, or conversely, where spread of adaptive alleles is likely to be slow and assisted gene flow may be necessary. Here, we apply this approach in a landscape genetic study of desert bighorn sheep (*Ovis canadensis nelsoni*) in the southwestern United States. We genotyped >850 individuals at neutral markers from three landscapes that vary with respect to habitat configuration and factors influencing gene flow, optimized landscape resistance models independently for these regions, and used genetic simulations to determine how differences among landscapes affect the capacity for spread of adaptive genetic variation within landscapes.

Desert bighorn sheep occupy some of the hottest and driest portions of the southwestern U.S, and their distribution is strongly limited by availability of reliable surface water and steep terrain to allow escape from predators [20]. Habitat configuration is highly variable across the subspecies’ range–linear and relatively continuous in some areas, but patchy in other areas–and presents an opportunity to explore the effects of habitat configuration on gene flow and natural selection. Other landscape characteristics also vary substantially across the subspecies’ range, including climate, vegetation, degree of anthropogenic development, and presence of major barriers to dispersal. Research on factors affecting gene flow or dispersal of desert bighorn sheep has been largely limited to a portion of the subspecies’ range in southern California and Nevada [14, 21], where landscape resistance models are currently being used to manage risks to connectivity from renewable energy development [22, 23].

Climate projection models predict increases in temperature and aridity in the southwestern U.S. in coming decades [24, 25], which could negatively impact bighorn sheep through decreasing water and forage availability or increasing heat stress. Bighorn sheep populations in the Mojave Desert have higher extinction probability [26] and lower genetic diversity [27] in hotter, drier low-elevation habitat than in cooler, wetter high-elevation habitat. Unlike many species that can respond to local climate change by shifting their spatial distribution either latitudinally or altitudinally to remain within their “bioclimatic envelope” [28–30], desert bighorn have limited ability to make such geographic shifts; they are habitat specialists that rely on steep and visually open escape terrain that often comprises a small percentage of the landscape that is discontinuously distributed, and they typically already occupy the most favorable (typically, wettest and coolest) portions of available habitat. Therefore, desert bighorn are likely to be strongly dependent on in-situ adaptation to deal with an increasingly inhospitable climate.

In this study, we explored two scenarios under which adaptive genetic variation could arise and spread throughout a region to facilitate climate change adaptation (hereafter, referred to simply as “scenarios”). In the first, a novel allele favored by selection was introduced at one location–for instance, via a mutation or the intentional translocation of individuals with a novel genotype–and subsequently spread outward from this origin point (hereafter, the “novel allele” scenario). In the second scenario, an allele that was already present throughout the region at low frequency became favored by selection due to a change in environmental conditions–for instance, a shift in climate regime–and subsequently increased in frequency throughout the region (hereafter, the “pre-existing allele” scenario). We simulated each of these scenarios (novel versus pre-existing allele) in three regions that differ with respect to habitat configuration and factors influencing landscape resistance for bighorn sheep, and we compared rates of adaptive allele spread among regions and scenarios while varying selection strength. We hypothesized that the rate of spread would be faster (1) in regions with more continuously distributed habitat because gene flow would be less limited by the need for high-cost dispersal between disjunct populations, (2) under the pre-existing allele scenario because local populations would be more likely to initially contain the adaptive allele and thus be less reliant on outside immigration to achieve adaptive allele spread locally, and (3) when selection for the adaptive allele was stronger.

## Materials and methods

Methods included the following major components: (1) collecting and genotyping DNA samples from individuals in three regions at 14–16 neutral microsatellite loci, (2) developing a suite of candidate landscape resistance models that describe how landscape variables influence gene flow, (3) using genetic data and least-cost path modeling to test the fit of candidate resistance models and identify an optimal model for each region, (4) simulating the spread of an adaptive allele in each region during 100 years of gene flow influenced by landscape resistance, with mate selection and dispersal determined as probabilistic functions of cumulative cost across optimized regional resistance surfaces, and (5) comparing results among regions for three selection strengths and two initial spatial distributions of the adaptive allele. We discuss each component in detail below.

### Study area

**Fig 1:**
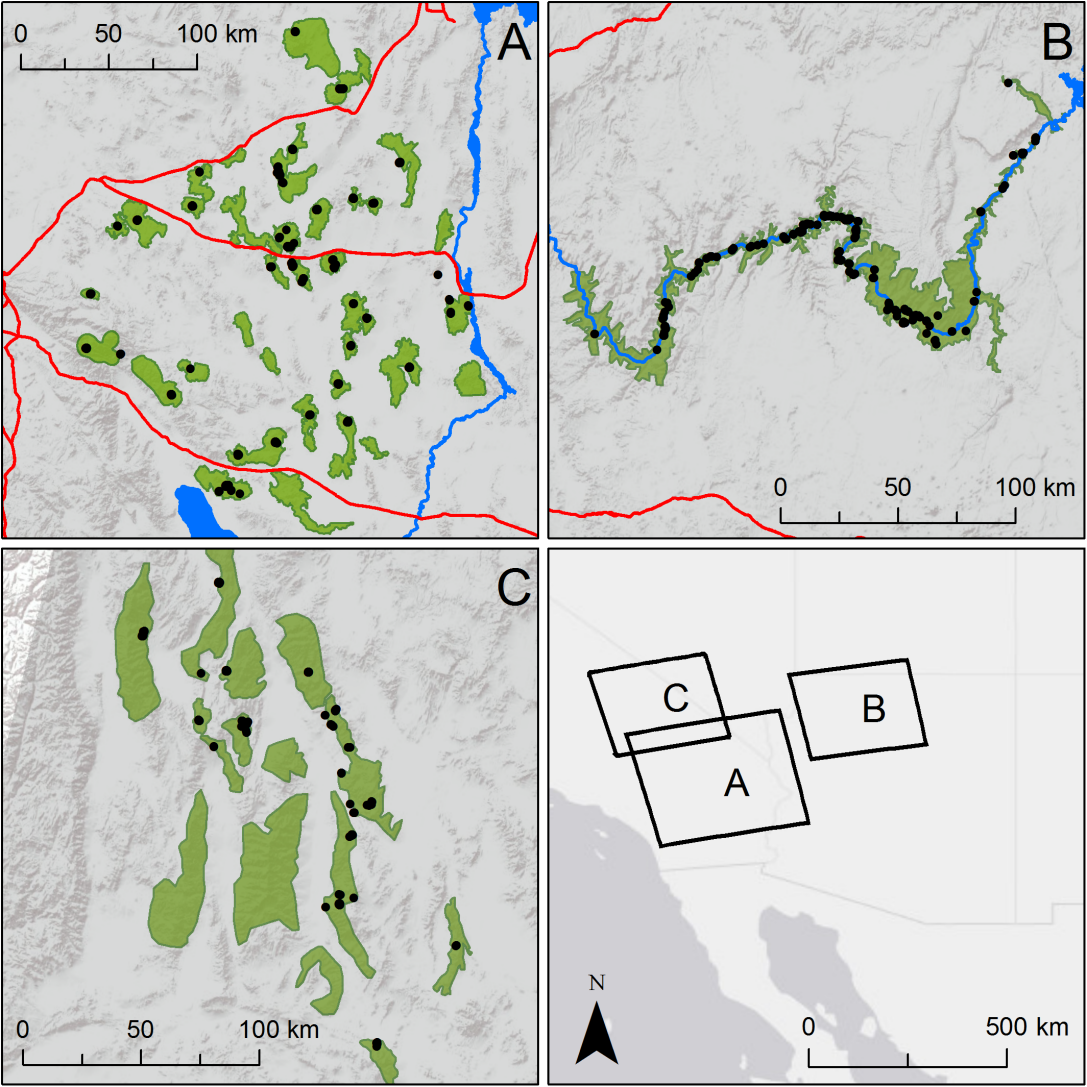
Genetic sampling locations. Black dots show where unique genotypes were sampled in each of three study regions: (A) southern Mojave, n = 378; (B) Grand Canyon, n = 252; (C) Death Valley, n = 225. Red and blue lines show major barriers to dispersal (highways and waterways, respectively). Green polygons show occupied bighorn habitat within which individuals were randomly located for CDPOP simulations of gene flow. Note that many black dots (individual locations) overlap at the scale of the map.

This study considers desert bighorn sheep populations in three regions of the southwestern U.S. that differ substantially in habitat configuration. The southern Mojave Desert region (hereafter, MOJA) of southeastern California and southern Nevada contains bighorn sheep habitat distributed in discrete mountain ranges within a matrix of less hospitable flats (Fig 1A). Populations in this region exhibit metapopulation structure, in which patches are linked by infrequent dispersal events [31, 32]. Human development within the region is limited, but three major interstate highways fragment the metapopulation, and ongoing renewable energy development threatens to further disrupt connectivity [33]. Two large protected areas, Mojave National Preserve and Joshua Tree National Park, are located within the region and are minimally impacted by human development.

The Grand Canyon region (hereafter, GRCA) in northern Arizona is dominated by the Colorado River flowing between Lake Mead and Lake Powell within Grand Canyon National Park (Fig 1B). Bighorn sheep are confined to the rugged terrain within the Grand Canyon and side canyons and avoid the surrounding plateau areas with poor escape terrain and visibility. The Grand Canyon is 445 river km long and 16 km wide on average within Grand Canyon National Park, so bighorn sheep habitat is relatively linear. GRCA is bordered by Glen Canyon and Lake Mead National Recreation Areas and habitat is continuous, but for the purposes of this analysis we consider only the GRCA section, which is more linear than adjacent habitat. Preliminary genetic analyses indicate strong genetic differentiation of individuals on opposite sides of the Colorado River, and weak differentiation within each side as a function of distance. Very little human development and few anthropogenic dispersal barriers exist within GRCA.

The Death Valley region (hereafter, DEVA) of the northern Mojave Desert along the California-Nevada border is centered on Death Valley National Park. Bighorn sheep populations occupy habitat patches that are relatively discrete and separated by flat, arid valleys, but are generally larger and more linear than in MOJA (Fig 1C); thus, DEVA represents an intermediate habitat configuration between MOJA and GRCA. Minimal human development and few anthropogenic dispersal barriers are present in the DEVA region.

### Genetic data

We obtained DNA mainly via non-invasive sampling of fecal pellets, and from a small number of tissue and blood samples from live captures, hunter kills, or carcasses found in the field. We used both newly-collected samples (2011–2013) from a larger project to assess genetic diversity around ten national parks in the southwest U.S. [34], and samples collected for previous studies. MOJA samples were collected during 2000–2004 [14, 27], while DEVA samples were collected in two phases: during 2003–2010 [35, 36] and during 2011–2013 to include areas previously omitted. Sampling in MOJA and DEVA was conducted primarily around water sources where bighorn sheep congregate during summer months. GRCA samples were collected during 2011–2013, and most samples were collected along the Colorado River and associated side canyons, with additional samples collected along trails and at observation points within the national park. UTM coordinates were recorded for all GRCA samples and for DEVA and MOJA samples collected after 2010, but only approximate locations (e.g., the name of a water source) were recorded for DEVA and MOJA samples from earlier sampling periods. We assigned coordinates to these earlier samples based on the geographic feature where they were collected.

Samples were analyzed using similar protocols in three genetics labs, corresponding to the three sampling periods: Oregon State University (OSU; 2011–2013), White Mountain Research Station (WMRS; 2003–2010), and University of California, Berkeley (UCB; 2000–2004). A total of 20 microsatellite loci were used across labs, but only 14–16 loci were used in each lab, with 10 loci common to all three labs (S1 Table). Descriptions of genotyping protocols for the two earlier periods (UCB and WMRS labs) can be found in Epps et al. [14] and Jaeger and Wehausen [35], respectively. We briefly describe the protocol for the most recent period (OSU lab, 2011–2013) below, but provide a more detailed description of DNA extraction, polymerase chain reaction (PCR) conditions, genotype calling and screening, and locus characteristics in S1 Appendix and S1 Table. We used a modified AquaGenomic Stool and Soil protocol (MultiTarget Pharmaceuticals LLC, Colorado Springs, CO) to extract DNA from material scraped from the surface of fecal pellets. We genotyped samples at 16 dinucleotide microsatellite loci in three multiplex PCRs of 4–6 loci using a Qiagen Multiplex PCR kit (Qiagen, Valencia, CA). We used an ABI 3730 capillary sequencer (Applied Biosystems [ABI], Foster City, CA, USA) to visualize PCR products and GENEMAPPER (version 4.1; ABI) to score genotypes. Each sample was amplified in at least three replicate PCRs to generate consensus genotypes. We used CERVUS version 3.0.3 [37] to identify duplicate genotypes and GIMLET version 1.3.3 [38] to estimate genotyping error rates (false allele occurrence rate and allelic dropout rate). We used GENEPOP version 4.2 [39] to test for deviations from linkage equilibrium and Hardy-Weinberg equilibrium.

### Genetic distances

We used the Bray-Curtis dissimilarity index (BC; [40]), equivalent to 1 minus the proportion of alleles shared between individuals, as a measure of inter-individual genetic distance to use in optimizing the landscape resistance models. BC is strongly correlated with and has provided similar performance to other individual-level genetic distance metrics (e.g., Rousset’s *a*_*r*_, PCA-based genetic distance) in previous studies [41–44]. We generated pairwise matrices of inter-individual genetic distance for each study area using the *ecodist* package [45] in R [46].

### Landscape variables

We considered seven variables that may affect bighorn sheep movement across the landscape (see S2 Table for information on geospatial data sources): (1) *Slope*. Bighorn sheep prefer steeper slopes that serve as escape terrain to evade predators [47]. (2) *Normalized Difference Vegetation Index (NDVI)*. This remotely-sensed measure of vegetation greenness is correlated with bighorn sheep diet quality [48, 49], and individuals could be more likely to move through areas offering better forage. We used a time-integrated NDVI (TIN) spatial dataset that estimates the total photosynthetic activity during the annual growing season. (3) *Anthropogenic development*. Bighorn sheep are intolerant of human activities in most cases [50] and may avoid permanently developed areas [20], although they can become habituated to human activities that are geographically predictable and non-threatening [51, 52]. (4) *Major roads*. Roads can be strong barriers to bighorn dispersal [14]. Genetic analyses [14, 21] and anecdotal evidence suggest that four-lane and fenced highways are rarely crossed by bighorn, while smaller, unfenced highways and roads are crossed frequently; thus, we considered only four-lane or fenced highways to be major roads. (5) *Distance to water*. The availability of permanent water sources is a key limiting factor for bighorn sheep populations [20] and may influence individuals’ ability or willingness to disperse through arid environments. We identified reliable water sources for bighorn sheep, including perennial streams, springs, seeps, lakes, reservoirs, and artificial guzzlers, and calculated the distance from each landscape cell to the nearest source. (6) *Forested areas*. Forested areas limit visibility and increase predation risk for bighorn sheep [53]. (7) *Water barriers*. Larger water features may serve as barriers to movement, as bighorn are thought to rarely cross high-volume rivers or reservoirs (e.g., Colorado River, Lake Powell). We used a combination of expert opinion, evidence from unpublished radio telemetry studies by NPS biologists, and a previous genetic study [35] to identify water barriers in the region. We did not include the major roads variable in our analysis for the DEVA or GRCA regions, or the water barriers variable in our analysis for the DEVA region, because these features were not present in these regions, respectively.

Geospatial data layers ranged in spatial resolution from 30 m to 250 m cells, but needed to be combined in a single-resolution, multivariate resistance layer. We used the *raster* package [54] in R to resample all layers to 3-arcsecond (approximately 100 m) cell resolution in order to meet computational limitations when calculating cost distances. Vector data layers were converted to raster using ArcGIS 10.1 [55].

### Candidate univariate surfaces

We used a combination of expert opinion and previous modeling studies to develop plausible alternative resistance parameterizations for each landscape variable to be tested with the genetic data. We included a large range of parameterizations to maximize the probability of bracketing the true resistance value. For continuous variables (slope, NDVI, distance to water), we modeled several possible relationships with landscape resistance (S2 Appendix), including linear relationships and concave-up and concave-down non-linear relationships (i.e., monotonic relationships in which the rate of change in resistance varies across the range of landscape variable values; S1 Fig). For slope, we also included Gaussian relationships (e.g., [44]) in which resistance was lowest at some intermediate slope value and increased as the slope value moved away from the optimum (S2 Fig), and breakpoint relationships in which slopes within an intermediate range were assigned a resistance value of 1, while slopes outside this range were assigned a single, higher resistance value. These relationships are plausible because shallow slopes may increase predation risk and very steep slopes could be too difficult for bighorn sheep to negotiate. For binary variables (anthropogenic development, major roads, forested areas, water barriers), we considered several possible ratios of resistance for the two types of cells (e.g., natural versus converted, or forested versus non-forested) by assigning the less resistant cell type a resistance value of 1 and assigning a range of resistance values for the more resistant cell type based on expert opinion. Additional detail on alternative parameterizations for continuous and categorical variables are in S3 Table and S4 Table, respectively.

### Cost distances

For each resistance surface (i.e., unique parameterization of a single landscape variable; *n* = 100 for DEVA, 110 for GRCA, 119 for MOJA), we used the *gdistance* package [56] in R to generate a pairwise matrix of inter-individual cost distances, calculated as the accumulated cost along the least-cost path [57] between sample locations for pairs of individuals. We used an individual-based rather than population-based approach for relating genetic distance and cost distance because it did not require defining populations a priori, and was therefore more appropriate in areas where bighorn sheep were continuously distributed (e.g., Grand Canyon). Recent studies have supported the use of individual-based approaches in landscape genetics [58, 59], even in cases where discrete populations exist [60].

### Resistance surface optimization

Mantel tests have been the standard approach for evaluating competing resistance surfaces in landscape genetic studies [5], but there is mounting evidence that they may not be appropriate or reliable for such applications [61–65]. We relied on an alternative approach that fits linear regression models using bootstrap sampling of independent pairs of individuals and has been successfully applied in several recent landscape genetic studies [66–70]. We used a two-phased approach [41] to optimize landscape resistance surfaces for each region: in the first phase, we tested sets of candidate resistance surfaces representing different resistance parameterizations of a single landscape variable and identified optimal univariate surfaces; in the second phase, we tested candidate multivariate surfaces including various subsets of the optimal univariate surfaces, as well as variants of the optimal univariate surfaces rescaled to have different maximum resistance values, and identified an optimal multivariate surface. This process was performed independently for each region, and we describe both phases in further detail below.

#### Univariate optimization

We first used a Mantel correlogram to estimate the Euclidean distance beyond which genetic distance and Euclidean distance were no longer correlated in each study region, and excluded all pairs separated by distances greater than this cutoff from our analysis; the purpose of this step was to remove pairwise comparisons that did not contribute useful information on the relationship between gene flow and environmental characteristics because of very long distances between individuals. Next, we used a pseudo-bootstrapping approach [66] to compare candidate resistance surfaces. This approach was similar to traditional linear regression, but accounted for the non-independence of pairwise data (in this case, genetic and cost distance matrices) by repeatedly selecting a random and independent subset of pairs from the dataset (i.e., each individual represented in only a single pairwise value). The maximum number of independent pairs that could be subsetted for each region was *N*/2, (where *N* was the number of individuals sampled in that region) because each individual could only be represented in one pairwise data point while maintaining statistical independence. In practice, the number of randomly sampled pairs was often slightly smaller than *N*/2 because some pairs were ineligible for selection due to the Euclidean distance threshold we imposed.

For each random subset, we fitted a linear regression model of genetic distance as a function of cost distance for each candidate resistance surface and calculated Akaike Information Criterion (AIC). We performed 10,000 iterations of this procedure, fitting all candidate models to the same random subset of pairwise data in each iteration and compared models using Akaike weight (the relative likelihood of a model, *exp*[-0.5*ΔAIC], divided by the sum of relative likelihoods of all models). We selected a top model from the candidate set using the median Akaike weight across iterations as our model selection criterion. A simple Euclidean distance surface (i.e., resistance surface with all cells having resistance value of 1) was included in the set of candidate surfaces for each variable to serve as a null model of isolation by distance (IBD). Because previous research has suggested that log-transforming cost-distances may improve linearity [64, 65, 71], we fit each model with both unlogged and log-transformed cost distances, and retained the version with the higher model *R*^2^.

#### Multivariate optimization

We generated a candidate set of multivariate resistance models by summing resistance values (on a cell-wise basis) for all possible combinations of landscape variables, using the optimized univariate resistance surface for each variable. Any variable for which the optimized univariate surface did not perform better than IBD (i.e., did not have higher median Akaike weight) was excluded from all candidate multivariate resistance surfaces. To allow for the possibility of interactions between variables (i.e., changes in the optimal resistance model for one landscape variable when effects of other landscape variables are included in a multivariate resistance model), we also created candidate multivariate models using univariate surfaces with the same shape of resistance curve as the best univariate surface, but with a different maximum resistance value. For instance, if the optimized univariate surface for the NDVI variable indicated a concave-down, negative relationship with a maximum resistance value of 50, we also created multivariate surfaces including concave-down, negative relationships with maximum resistance values of 10 and 100 for NDVI. We could not test all possible combinations of univariate models because allowing all univariate model parameters to vary for each landscape variable in multivariate models would have resulted in an excessive number of multivariate models. Other methods have been proposed to maximize the amount of the multivariate hypothesis space explored (e.g., Shirk et al. 2010), but all methods are constrained to some extent by computational limitations. We compared multivariate surfaces using the bootstrap AIC approach described above. Because four or fewer landscape variables were more informative than the null model of IBD in each region, the number of multivariate models remained reasonable.

### Simulation of adaptive allele spread

After identifying the best landscape resistance model for each study region using landscape genetic analysis of neutral markers, we used the computer program CDPOP v1.2 [72] to simulate gene flow and natural selection in each of our study regions. CDPOP simulates dispersal and mating of individuals across a landscape resistance surface, allowing the user to define the initial genetic structure, spatial distribution of individuals, dispersal characteristics, and life history traits of the population. Natural selection is incorporated by allowing offspring mortality rate to vary as a function of individual genotype linked to environmental associations. We simulated selection at a single biallelic locus with an adaptive allele *A* and a neutral allele *a*. We tested three different strengths of selection for the adaptive allele: a 10 percent (“weak selection”), 20 percent (“moderate selection”), or 30 percent (“strong selection”) increase in offspring survival of the *AA* genotype relative to the *aa* genotype. We assumed additive dominance, whereby survival of the *Aa* genotype was intermediate (*h* = 0.5) to the two homozygotes.

#### Initializing individual locations and genotypes for simulation

We used maps of occupied desert bighorn sheep habitat provided by state wildlife agencies to assign individual locations, which remain fixed throughout simulations in CDPOP. Individuals were randomly placed within occupied habitat at a constant density of 0.2 individuals/km^2^ in each region, resulting in 1,684 individuals for DEVA, 624 for GRCA, and 1,576 simulated individuals for MOJA. We arrived at this density by summing population size estimates for the MOJA and DEVA regions (based on the most recent available information, e.g., Epps et al. 2003) and dividing by the total area of occupied habitat within these two regions; a population estimate was unavailable for GRCA, so we assumed that the average bighorn sheep density in the other regions was a suitable estimate for GRCA. The assumption of constant density of individuals within and across regions was preferable to using actual population sizes because (1) population information was unavailable or outdated in many areas; (2) bighorn sheep population sizes can change dramatically over short time scales, especially in metapopulation systems such as the Mojave Desert, or in the event of a disease outbreak; thus, current population estimates may only remain accurate for a short portion of the simulation time frame; and (3) we wanted to investigate the effects of differences in landscape configuration and resistance among regions without the variation introduced by differences in local population density.

For the novel allele scenario, we initialized genotypes with allele frequencies of 0.01 and 0.99 for the adaptive (*A*) and neutral (*a*) alleles, respectively, in each regional population. We selected a single individual near the center of each region and identified the closest two percent of neighboring individuals in the landscape, based on cost distance. Among this subset of individuals, we randomly assigned half of the pooled alleles to be the *A* allele, and all remaining alleles within the region to be the *a* allele, creating a small cluster of *AA*, *Aa* and *aa* genotypes at Hardy-Weinberg equilibrium frequencies within a regional population that was otherwise homozygous for the *aa* genotype. These clusters of adaptive alleles were approximately 15 km in diameter in all three regions, and spanned portions of two populations each in DEVA and MOJA, and a small portion of the single continuous population in GRCA. We used this cluster strategy rather than initializing simulations with a single copy of an adaptive allele, as would occur immediately following a mutation, because a single allele would quickly be removed from the population by genetic drift in most cases, even when selection was strong. Thus, this scenario might exemplify examining spread of a local adaptation or variant. For the pre-existing allele scenario, simulating a change in selective coefficient for an allele already present at some frequency across the region, we initialized genotypes with regional allele frequencies of 0.05 and 0.95 for the *A* and *a* alleles, respectively, and each allele randomly distributed among individuals in the region.

#### Simulation parameters

We simulated gene flow for 100 years following the initiation of genotypes, with 50 Monte Carlo replicates for each combination of selection strength (none, weak, moderate, strong) and scenario (novel allele or pre-existing allele). Mating and dispersal movements followed an inverse-square function of cost distance. Although bighorn sheep exhibit sex-biased dispersal [47], preliminary simulations using smaller dispersal thresholds for females caused populations to decline across the study area over the simulation period; thus, to achieve relatively stable population dynamics necessary to examine how adaptive allele spread varied among regions, we used the same dispersal thresholds for males and females. To standardize cost distances among regions, we added 2 to each cell value in the optimized DEVA resistance surface so that the cost value of the least resistant cell type was constant across regions; this was necessary because multivariate surfaces were created by summing three univariate surfaces with a minimum value of 1 for GRCA and MOJA, but only a single univariate surface for DEVA.

We first ran simulations with a maximum dispersal threshold of 534,861 cost units, the cost distance beyond which genetic distance and cost distance were no longer correlated within the GRCA region; this was the smallest of such estimates for the three regions, using the best multivariate resistance models to estimate cost distance. Because maximum dispersal distance of bighorn sheep has not been precisely estimated and could influence the relative rate of spread of adaptive alleles, we repeated all simulations at ½ the original dispersal threshold (267,430 cost-units) and at twice the original dispersal threshold (1,069,722 cost units) to bracket a range of likely dispersal thresholds; we hereafter refer to these threshold values as “low”, “medium”, and “high” dispersal thresholds. These cost distance thresholds correspond to Euclidean distances ranging between 2.4 and 13.6 km if individuals traveled through average-resistance terrain in each region; however, actual distances traveled in simulations could be much further than this because we assumed individuals traveled along least-cost paths. We allowed males but not females to mate with replacement in order to approximate the polygynous mating system of bighorn sheep. The population included 17 annual age classes, with age-specific mortality and fecundity rates estimated from the literature [20, 47, 73–75]. Each mating event resulted in a single offspring, as twinning is rare in bighorn sheep [76]. We set mutation rate to zero, given the short time frame of the simulations (<15 generations).

#### Quantifying adaptive allele spread

We calculated the mean adaptive allele frequency (hereafter, *f*_*A*_) at every year by averaging results from the 50 MC replicates for each combination of selection strength and scenario. We plotted 95% confidence bands for *f*_*A*_ in each region as a function of time and compared confidence bands for differences in the rate of adaptive allele spread among regions.

## Results

### Genetic data

We genotyped 225 unique individuals from DEVA, 252 from GRCA, and 378 from MOJA. False allele occurrence rate was zero in all regions, and allelic dropout rate averaged 4.1 percent across loci and regions. We observed deviations from Hardy-Weinberg equilibrium or linkage equilibrium in a number of populations within the three regions; however, no locus (for HWE) or pair of loci (for LE) was consistently out of equilibrium across populations, suggesting that these deviations most likely resulted from population substructure rather than non-neutral loci or non-independent loci. We therefore retained all loci in subsequent analyses.

### Univariate optimization

The number of randomly sampled pairs ranged among iterations from 109 to 112 for DEVA, 123 to 125 for GRCA, and 185 to 188 for MOJA. Landscape variables that were supported by univariate optimization (i.e., that had higher Akaike weight than the null model of isolation by Euclidean distance) differed among regions (Table 1). Slope was supported in all three regions, and was the strongest univariate predictor in DEVA and MOJA, as indicated by median *R*^2^. A Gaussian slope model was preferred over a linear model or break-point model, although the parameters of the Gaussian model (optimal slope, maximum resistance value) differed between regions. Presence of water barriers was associated with increased resistance to gene flow and was the variable with the greatest explanatory power in GRCA, but was not supported in the remaining two regions. Similarly, presence of major roads was associated with increased resistance and was an important variable in MOJA but not in DEVA or GRCA. Distance to water (positively associated with resistance) was supported in DEVA and GRCA, and NDVI (negatively associated with resistance) was supported in DEVA and MOJA, but these variables only explained slightly more variation than Euclidean distance in these regions. Forested areas and anthropogenic development were not supported in any region and were excluded from multivariate optimization. Models with unlogged cost distances were preferred for all variables in all regions, with the exception of the isolation by distance model in GRCA.

**Table 1:** Optimized univariate resistance models for each region.

Region	Landscape variable^a^	Optimal resistance surface	Median *R*^2^
DEVA			
	Slope	Gaussian (r_max_ = 100, x_opt_ = 50, x_sd_ = 20)	0.151
	Distance to water	Monotonic positive (r_max_ = 50, r_exp_ = 4)	0.132
	NDVI	Monotonic negative (r_max_ = 10, r_exp_ = 0.25)	0.130
	Euclidean distance	—	0.129
GRCA			
	Water barriers	Ratio (1,000)	0.173
	Slope	Gaussian (r_max_ = 10, x_opt_ = 50, x_sd_ = 20)	0.062
	Distance to water	Monotonic positive (r_max_ = 50, r_exp_ = 1)	0.063
	Euclidean distance^b^	—	0.050
MOJA			
	Major roads	Ratio (100)	0.227
	Slope	Gaussian (r_max_ = 10, x_opt_ = 40, x_sd_ = 20)	0.200
	NDVI	Monotonic negative (r_max_ = 100, r_exp_ = 0.25)	0.186
	Euclidean distance	*—*	0.173

^a^ Variables not included in the table did not outperform the null model of isolation by distance (i.e., had lower median Akaike weight) and were excluded from further analysis.

^b^ Cost distance was log-transformed only for the GRCA Euclidean distance model because untransformed cost distances produced higher model *R*^2^ for all other resistance models.

### Multivariate optimization

The optimized multivariate model for GRCA included slope, water barriers, and distance to water. For MOJA, the optimized multivariate model included slope, roads, and NDVI. The univariate slope model was preferred over all multivariate models for DEVA. Table 2 provides details on the relationships between each variable and resistance to gene flow for the optimized multivariate model in each region. Resistance values associated with variables in optimized univariate models sometimes differed from those in optimized multivariate models; for instance, water barriers were assigned a resistance of 1,000 in the best univariate model for GRCA, but a value of 5,000 in the best multivariate model.

**Table 2:** Optimized multivariate resistance model for each region.

Region	Model	Median *R*^2^
DEVA^a^	*slope* (Gaussian: r_max_ = 100, x_opt_ = 50, x_sd_ = 20)	0.152
	*water barriers* (ratio = 5,000) +	
GRCA	*slope* (Gaussian: r_max_ = 10, x_opt_ = 50, x_sd_ = 20) +	0.177
	*distance to water* (positive: r_max_ = 100, r_exp_ = 1)	
	*roads* (ratio = 5,000) +	
MOJA	*slope* (Gaussian: r_max_ = 50, x_opt_ = 40, x_sd_ = 20) +	0.259
	*NDVI* (negative: r_max_ = 10, r_exp_ = 0.25)	

^a^ The univariate slope model outperformed all multivariate models in the DEVA region.

Explanatory power of optimized multivariate models was relatively low as measured by model *R*^2^: cost distances explained less than a quarter of the variation in genetic distances in all regions. The multivariate models for GRCA and MOJA represented only a modest increase in explanatory power over the best univariate model. In the DEVA and MOJA regions, Euclidean distance alone explained at least two thirds as much variation as the best multivariate resistance model. However, distance was a less powerful predictor of genetic differentiation in GRCA, where the barrier effect of the Colorado River explained the majority of variation in genetic distances (Tables 1 and 2).

### Simulations

Genetic simulations revealed differences in the spread of adaptive genetic variation among regions for many combinations of selection strength, dispersal threshold, and scenario. Under moderate to strong selection, we observed higher frequencies of the adaptive allele in the GRCA region, which had the most continuous distribution of habitat, than in MOJA or DEVA, which had low to intermediate habitat continuity relative to GRCA. Under most combinations of selection strength, dispersal threshold, and scenario that we tested, differences in *f*_*A*_ that emerged between DEVA and MOJA were also consistent with our hypothesis of faster spread of adaptive alleles in more continuous landscapes; however, because DEVA and MOJA were much more similar with respect to habitat continuity than either was to GRCA, differences between DEVA and MOJA were relatively small.

Adaptive allele frequency (*f*_*A*_) was positively associated with selection strength and dispersal threshold in both simulation scenarios. We observed greater increase in *f*_*A*_ in landscapes with more continuously distributed habitat under both scenarios. Relative differences in *f*_*A*_ among regions (i.e., the ratio of *f*_*A*_ for two regions at a given point in time) tended to be larger under the novel allele scenario.

#### Novel allele scenario

**Fig 2:**
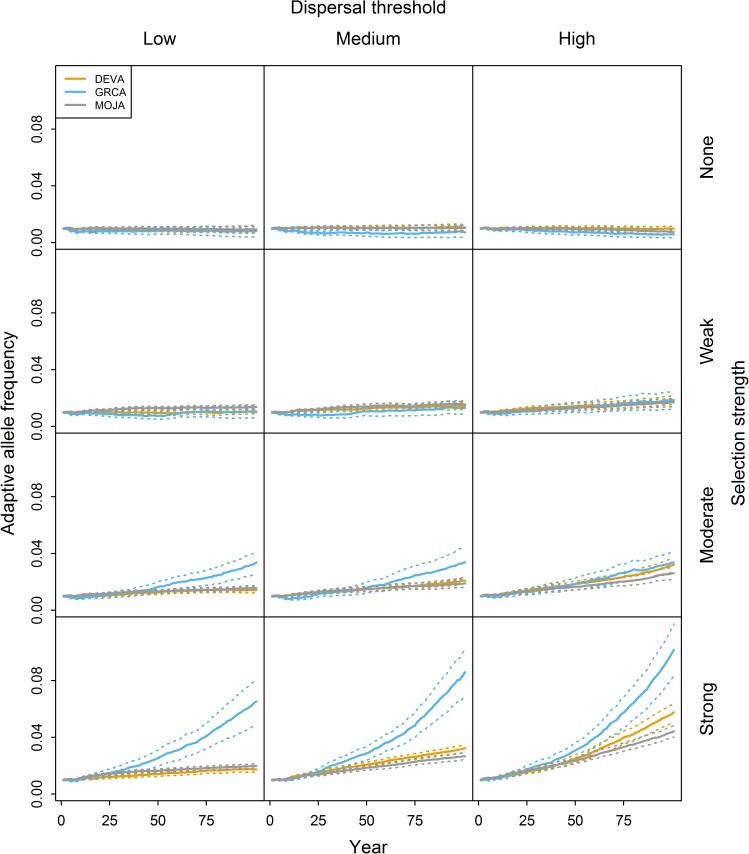
Simulated change in adaptive allele frequency through time under the novel allele scenario. Results are shown for multiple selection strengths and dispersal thresholds in the DEVA, GRCA, and MOJA regions. Simulations were initiated with a small cluster of adaptive alleles at the center of each region. Rows and columns correspond to selection strengths and dispersal thresholds, respectively. Solid and dashed lines represent means and 95 percent confidence limits, respectively, from 50 MC replicates per region.

Under the novel allele scenario, relatively small increases in *f*_*A*_ were observed over the simulation period (Fig 2). Even under strong selection, *f*_*A*_ remained below 0.12 after 100 years. The effect of landscape (i.e., difference in *f*_*A*_ among regions) was slow to emerge (25–50 years under most conditions) and was more pronounced when selection was stronger and maximum dispersal threshold was larger. GRCA clearly exhibited higher *f*_*A*_ than DEVA and MOJA for all dispersal thresholds when selection was moderate or strong. Differences in *f*_*A*_ among DEVA and MOJA were only evident when the high dispersal threshold was used and selection was moderate or strong; for all other combinations of dispersal threshold and selection strength, there was no appreciable difference in *f*_*A*_ between DEVA and MOJA. Where differences were evident, *f*_*A*_ tended to be higher for DEVA than for MOJA, consistent with our hypothesis of faster spread of the adaptive allele in regions with more continuously distributed habitat.

#### Pre-existing allele scenario

**Fig 3:**
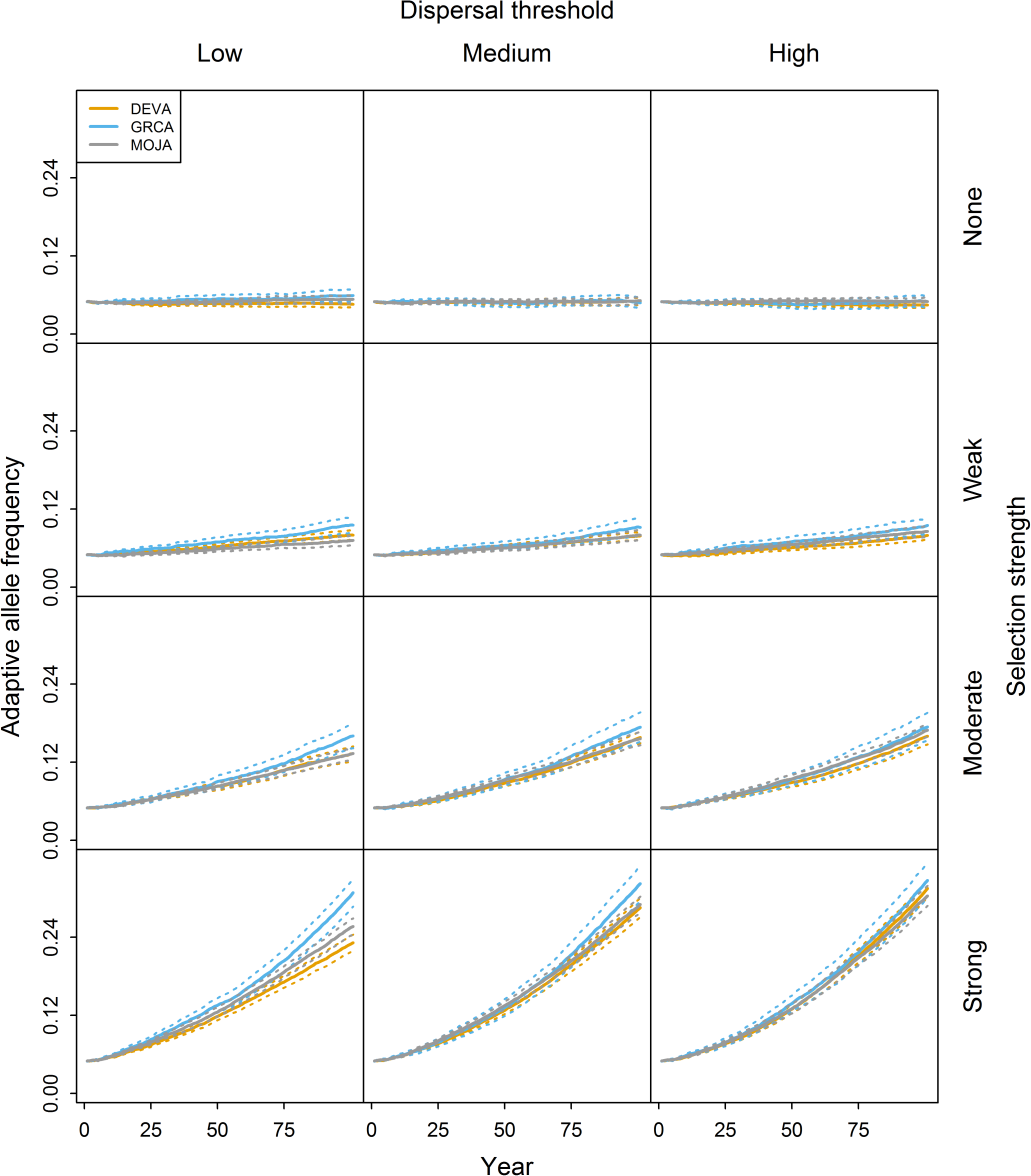
Simulated change in adaptive allele frequency through time under the pre-existing allele scenario. Results are shown for multiple selection strengths and dispersal thresholds in the DEVA, GRCA, and MOJA regions. Simulations were initiated with the adaptive allele randomly distributed throughout each region at 5 percent frequency. Rows and columns correspond to selection strengths and dispersal thresholds, respectively. Solid and dashed lines represent means and 95 percent confidence limits, respectively, from 50 MC replicates per region.

We observed much greater increases in *f*_*A*_ over time under the pre-existing allele scenario than the novel allele scenario, with *f*_*A*_ reaching nearly 0.35 by year 100 under some conditions (Fig 3). For all combinations of selection strength and dispersal threshold that produced differences among regions, *f*_*A*_ was higher for GRCA than the other regions. When selection was weak to moderate, DEVA and MOJA exhibited similar increases in *f*_*A*_. However, when selection was strong, the effect of landscape depended on dispersal threshold: with the low dispersal threshold, *f*_*A*_ was actually higher in MOJA than DEVA, while *f*_*A*_ was approximately equal for the two regions with the medium or high dispersal threshold.

## Discussion

We developed landscape resistance models for desert bighorn sheep in three regions with different habitat configuration and factors affecting resistance to gene flow, and found that these differences among landscapes influenced the spread of adaptive genetic variation in subsequent genetic simulations. However, not all combinations of selection strength and dispersal threshold resulted in clear differences among landscapes with respect to adaptive allele spread. Observed differences among regions in adaptive allele spread were generally consistent with expectations of faster spread when landscapes exhibited more continuously distributed habitat and when a pre-existing allele (i.e., standing genetic variation) rather than a novel allele (i.e., mutation) served as the source of adaptive genetic variation.

### Resistance modeling

Our resistance model optimization suggested that slope and strong dispersal barriers, including major water bodies and interstate highways, were the dominant landscape factors influencing gene flow for desert bighorn sheep. These results are consistent with previous research demonstrating strong effects of slope and highways on bighorn gene flow in the Mojave Desert [14, 21], and also complement earlier analyses suggesting that that major water barriers (i.e., the Colorado River) currently limit gene flow for bighorn sheep [35]. A Gaussian model in which both very low and very high slopes have high resistance was supported, suggesting that some areas of our study regions are actually steep enough to prevent movement by bighorn sheep. Interestingly, the effect of slope appeared to vary by region with respect to the optimal slope and the maximum resistance associated with slope; for instance, slope was assigned a maximum resistance of 100 in DEVA but only 10 in GRCA. It is not clear why sub-optimal slopes would have presented a greater obstacle to bighorn sheep in DEVA than GRCA, but the distribution of favorably sloped terrain within each region may have influenced this result. Habitat in GRCA comprises one highly continuous patch of favorably-sloped terrain, the Grand Canyon, allowing bighorn sheep to travel long distances with limited exposure to highly resistant slopes (with the exception of some near-vertical canyon walls that cannot be traversed); in contrast, even short-distance travel between neighboring patches in MOJA typically requires traversing low-slope, high-resistance areas. Thus, the effect of slope may appear to be weaker in GRCA simply because dispersal is less limited by slope, at least for horizontal movements within the canyon.

This example illustrates an important and well-known limitation in landscape genetic analyses: features that influence gene flow, but are not highly variable within the landscape, are often not supported in landscape resistance models [77]. This limitation may also explain why we failed to detect an effect of some landscape variables that are known to strongly influence movement behavior of bighorn sheep (e.g., forested areas, anthropogenic development). The vast majority of each region we examined comprised natural cover types, with anthropogenic development limited to a few peripheral areas, so it may not have been possible to detect an effect of development, even if it strongly influenced dispersal for those few individuals that occupy habitat close to development. Landscape genetic effects are also difficult to detect in highly connected landscapes [65, 78], where individuals are largely able to avoid traversing through resistant features. This scenario may apply, for instance, to forested areas in the GRCA region, which occur almost exclusively on plateaus surrounding the Grand Canyon–that is, in areas of low slope that are poor bighorn sheep habitat and can be avoided by traveling within the canyon. These examples suggest that spatial extrapolation of locally-developed resistance models could lead to omission of important factors affecting dispersal and gene flow, and researchers wishing to apply resistance models in new areas should recognize this limitation.

Euclidean distance explained at least two thirds as much of the genetic differentiation among individuals as the final optimized model in DEVA and MOJA, suggesting that isolation by distance is strong in these regions. However, distance was a relatively poor predictor of genetic differentiation in GRCA, which was likely driven by the unique juxtaposition of suitable habitat and a major barrier in GRCA: the steeply sloped Grand Canyon provides a long, narrow, and continuous habitat patch for bighorn sheep, but is bisected by the Colorado River running through the bottom of the canyon and serving as a strong barrier to movement. Thus, two individuals on opposite sides of the river that were sampled only hundreds of meters apart may have less chance of mating than two individual on the same side of the river that were sampled tens to hundreds of kilometers apart.

### Simulating selection

The simulated rates of spread of adaptive genetic variation in our three study regions largely supported our expectation of faster spread in landscapes with more continuously distributed habitat. In fact, our results may actually underestimate differences among the regions because population density was assumed to be constant through time and between habitat patches in CDPOP simulations, but densities tend to fluctuate in space and time in real populations. Such fluctuations are likely to be most dramatic in patchy landscapes like the MOJA region that exhibit metapopulation dynamics [31, 32]. Theoretical models suggest that extinction and recolonization reduce fixation probability for beneficial alleles [79, 80], and that probability of fixation of beneficial alleles decreases when reproductive success varies among demes [81]. These effects should be stronger in patchier systems and reinforce the differences we observed between regions.

The effects of selection strength and dispersal threshold on *f*_*A*_ were generally consistent with our expectations: *f*_*A*_ increased faster when selection was stronger or dispersal threshold was larger. The effect of dispersal distance on *f*_*A*_ values was much smaller than the effect of selection in most cases. Our high dispersal threshold was four times larger than our low threshold, but *f*_*A*_ values tended to be only marginally higher using the high threshold. We suspect that this is because we used an inverse square dispersal function resulting in very common short-distance movements and very rare long-distance movements, such that increasing the maximum dispersal distance may have had only a small influence on the average distance moved by an individual. Exploring dispersal functions was beyond the scope of this study and should be left for future theoretical work.

In some cases, we observed interesting interactions between selection strength and dispersal threshold. The degree to which differences among regions arose during simulations depended on the combination of selection strength and dispersal threshold considered, with regional differences quite pronounced for some combinations and minimal for others. In a few cases, the choice of dispersal threshold even reversed the conclusion regarding the relative spread of an adaptive allele in the two relatively patchy regions; for instance, under strong selection in the pre-existing allele scenario, *f*_*A*_ increased faster for DEVA than MOJA with the medium dispersal threshold, but the opposite was true with the low dispersal threshold. This interplay between dispersal threshold and selection strength is a potentially complex topic that warrants further investigation.

**Fig 4:**
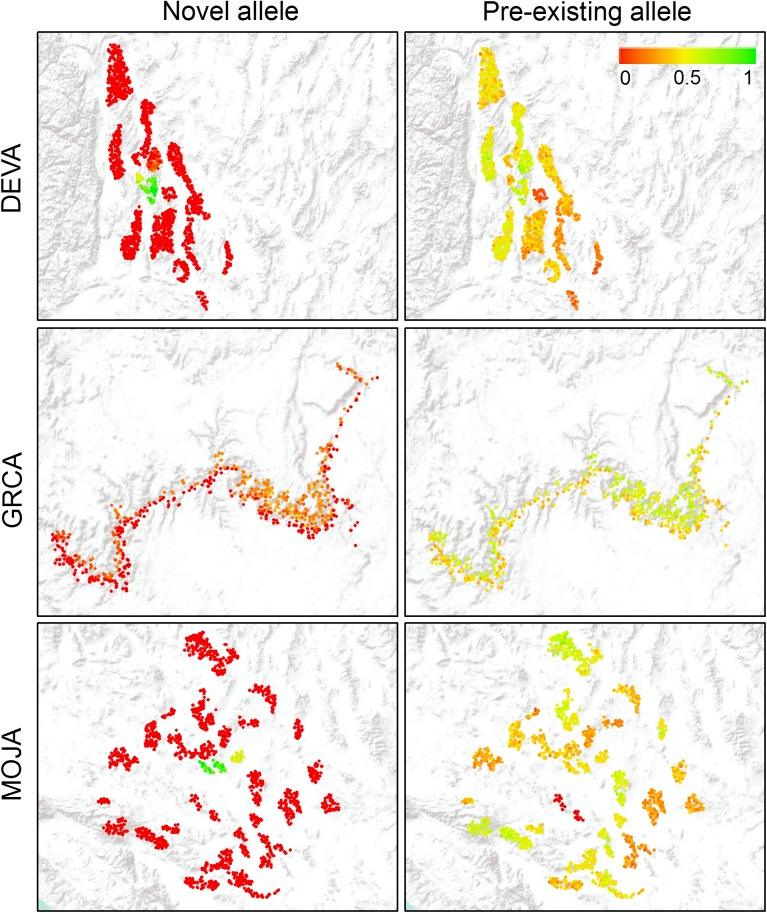
Simulated spatial spread of adaptive allele in different regions over 100 years. Colored dots represent individual locations, and black polygons represent national park boundaries. Color gradient reflects proportion of MC replicates in which adaptive allele is present (≥1 copy) in each individual location at year 100 for each region and each scenario, assuming strong selection and medium dispersal threshold.

We observed large differences in the trajectory of *f*_*A*_ under the novel allele and pre-existing allele scenarios. This may be partially due to the fact that initial allele frequencies differed between the scenarios (0.01 versus 0.05), as they were intended to simulate different processes by which adaptive alleles could be introduced and spread throughout a landscape. However, examination of the spatial spread of the adaptive allele under each scenario suggests limitations on inter-patch dispersal imposed by landscape resistance were also likely responsible for the difference between scenarios. As an example, Fig 4 shows the spread of the adaptive allele across each region by year 100 under strong selection and moderate dispersal threshold for both scenarios. Under the novel allele scenario, where the adaptive allele was initially present in only one location, spread was limited to nearby patches in DEVA and MOJA, although *f*_*A*_ within those patches was close to 1. This reflects the presence of high-resistance terrain (e.g., desert flats, possibly with roads) separating patches and making inter-patch dispersal events rare. GRCA, with its highly continuous habitat, exhibited much greater geographic spread, although limited to the side of the Colorado River on which the adaptive allele was initially present. Under the pre-existing allele scenario, however, spread of the adaptive allele was much more extensive in all three regions. This occurred because the adaptive allele was initially present in all patches within each region, and thus increases in *f*_*A*_ could occur solely through intra-patch dispersal that did not require traversing high-resistance terrain. This also explains why regional differences in *f*_*A*_ were much smaller under the pre-existing allele scenario: habitat patchiness played much less of a limiting role when inter-patch dispersal was not needed to introduce the adaptive allele to new populations.

Given the major differences between the two scenarios, it is helpful to consider the circumstances that could lead to each scenario and the implications of each for adaptation. Novel alleles arise naturally in populations through mutation, but the likelihood of such a mutation giving rise to regional adaptation to climate change or some other stressor is probably low because (1) mutation rates are generally small, so novel alleles should arise infrequently [82], especially in large mammals with long generation times; (2) most mutations are selectively neutral or deleterious [83]; and (3) even those mutations that are favored by selection are often lost through genetic drift [84], particularly in small populations or metapopulations with high turnover rates (e.g., bighorn sheep in patchier landscapes). Therefore, a more likely source of novel alleles is the intentional translocation of one or more individuals from another region that are known to possess favorable traits that could improve survival or reproduction in the target area; for instance, individuals adapted to hotter, drier conditions in a different part of a species’ range, or those found to have disease-resistant genotypes, could be translocated. In contrast, the pre-existing allele scenario presupposes that standing genetic variation can provide the source material for adaptation; that is, alleles that exist at low frequency in the population and are maintained by a balance of recurrent mutation, selection, and drift become more favorable as biotic or abiotic environmental conditions change [85]. Standing genetic variation should lead to faster evolution than is possible with novel mutations, as well as fixation of more alleles with smaller effect and spread of more recessive alleles [85]; recent case studies (e.g., [86]) have demonstrated that standing genetic variation can facilitate rapid adaptation to novel conditions. We initialized pre-existing adaptive alleles randomly across each region in our simulations, but it may be more realistic to think of clinal variation associated with an environmental gradient (e.g., temperature or precipitation), or variation that is distributed unevenly across populations due to differences in connectivity or population size.

We limited our simulations to 100 years because we sought to explore adaptive changes occurring on a temporal scale relevant to conservation and management. Previous research in our study system showed that the effects of barrier construction changed genetic population structure in fewer than 50 years [14]. A brief investigation of changes in allele frequency over a longer time frame (1,000 years) suggested that the patterns that begin emerging by year 100 continue to manifest over longer time periods, and that the shorter simulations appear to capture the differences in trends that lead toward different asymptotic allele frequencies (S3 Fig). However, simulations over longer time frames may reveal additional insights into landscape effects on patterns of adaptive variation.

We explored a very simplistic selection model in which fitness was dependent upon an individual’s genotype at a single locus exhibiting additive dominance. However, most quantitative traits are determined by multiple genes [87, 88], and fitness may depend on non-additive effects of alleles at multiple loci (i.e., epistatic effects; [89]). In addition, we assumed that the adaptive allele in our simulations was universally favored, independent of the environmental characteristics experienced by each individual (i.e., flat selection surfaces). This may be appropriate for some types of adaptive variation (e.g., genes linked with pathogen resistance), but many genes control traits that are directly linked to environmental characteristics (e.g., thermal tolerance limits), and selection will not act in a spatially homogeneous manner if the landscape is heterogeneous with respect to the environmental characteristic of interest. Simulation studies with more realistic selection models will be necessary to fully understand how differences among landscapes contribute to the spatial distribution of adaptive genetic variation. Nevertheless, we have demonstrated an approach that can serve as a starting point for future work incorporating greater ecological and evolutionary complexity and realism.

### Implications for conservation and management

The results of our resistance modeling have important implications for management of connectivity among desert bighorn sheep populations. Beyond the simple distance between individuals, slope and major dispersal barriers (highways and large waterways) were the primary determinants of landscape connectivity. From a conservation perspective, this may be encouraging because the slope of terrain should be negligibly influenced by climate change or anthropogenic development, and barriers to bighorn sheep dispersal can often be mitigated through construction of crossing structures (e.g., [90]). NDVI and distance to water were also included in one regional multivariate model and may be more strongly linked to climate change, as forecasted increases in aridity in the southwest U.S. could result in loss of surface water sources and reduction in forage quantity or quality; however, these variables explained a much smaller proportion of the variation in inter-individual genetic distance than did slope and barriers. It is possible that the effects of these variables on gene flow could increase in the future as climatic conditions change, particularly if threshold values not currently observed in our study regions are exceeded.

Our gene flow simulations suggested that the spread of adaptive genetic variation is likely to occur slowly for desert bighorn sheep, even in places where connectivity has not been compromised and natural selection strongly favors an adaptive allele. In patchy systems like the MOJA region (and to a lesser extent, the DEVA region) where many populations are small, genetic drift can overwhelm selection [91, 92]. The spread of adaptive variation was especially slow for the novel allele scenario, where even an allele that was strongly favored by selection and already present throughout a region at low frequency took 25–50 years to noticeably increase in frequency. Increase in *f*_*A*_ was considerably faster for the pre-existing allele scenario, but even after 50 years of strong selection, *f*_*A*_ remained below 0.2 in all regions. Furthermore, this was probably optimistic because we initialized the pre-existing allele scenario assuming that the adaptive allele was already distributed across the entire region and present in all populations, which is unlikely to be true in real-life situations. These results highlight the need to maintain standing genetic diversity through conservation measures that promote stable, well connected populations. The GRCA region may be particularly important in this regard because of its large population size and continuously distributed habitat, which could allow for both the maintenance of pre-existing diversity and the rapid spread of adaptive alleles.

Using equal movement thresholds for males and females was necessary to achieve stable population dynamics in our simulations, but was not biologically realistic because bighorn populations tend to exhibit sex-biased dispersal [47]. Our estimated distance thresholds were based on observed relationships between cost distance and genetic distance, and likely reflected dispersal (and subsequent gene flow) of males over longer distances. Using equal distance thresholds for the sexes may therefore have led to unrealistically long dispersal events by females relative to males in our simulations. However, we believe this discrepancy between simulated and real-life movement behavior had limited effect on our main conclusions because any resulting biases would have applied equally to the three study regions, and we also explored the sensitivity of our results to changes in dispersal threshold. We observed generally slow rates of adaptive allele spread in our simulations, and with shorter female movements, the rate of spread could be even slower.

The slow pace of selection is partly a reflection of the relatively long generation time of bighorn sheep; the 100-year period of our simulations may seem long from a wildlife conservation and management perspective, but is exceedingly short from an evolutionary perspective, representing fewer than 20 bighorn sheep generations. This has two important ramifications. First, relying on existing genetic variation and natural gene flow to promote adaptation to climate change by desert bighorn sheep may not be a realistic conservation option given the rapid forecasted rate of change. Second, if the introduction of novel adaptive alleles to a region via translocation is desired, in may be necessary to target multiple locations, particularly in areas such as MOJA and DEVA with distinct subpopulations, to achieve sufficient regional spread within a time frame relevant to conservation.

Our genetic simulations assumed that habitat configuration did not change during the simulation period. Because bighorn sheep habitat is strongly limited by topography and surface water availability, major geographic shifts in response to climate change are unlikely. However, if forage quality declines or water sources dry up due to increasingly arid climate conditions, currently occupied habitat could become unsuitable in some areas. Future research could explore how projected climatic changes are likely to affect habitat configuration and the potential for adaptive allele spread in the study regions.

### Applications

The framework we have presented here–combining optimization of resistance models and genetic simulations–could be applied by conservationists and managers in a number of ways to help species cope with climate change and other threats to population persistence. It could be used to identify the most effective locations in a region to translocate individuals possessing favorable genotypes with respect to traits such as thermal tolerance or disease resistance, with the goal of maximizing the subsequent spread of adaptive alleles. This approach should not necessarily be limited to large mammals, or even animals; for instance, outplanting resistant tree stock has become a standard practice for restoring forests affected by introduced pests and pathogens [93], and our approach could potentially improve the efficiency of outplanting programs that can target only a limited number of areas. As our understanding of climate-linked genetic variation improves through advances in population genetics methods and technology [94], so too should our ability to accurately model and predict the spread of adaptive diversity.

## Supporting information

S1 Appendix Genetic laboratory methods. (PDF)

S2 Appendix Resistance model equations. (PDF)

S1 Dataset Individual genotypes and sampling coordinates for Death Valley region. (CSV)

S2 Dataset Individual genotypes and sampling coordinates for Grand Canyon region. (CSV)

S3 Dataset Individual genotypes and sampling coordinates for southern Mojave region. (CSV)

S1 Fig Monotonic resistance relationships. (PDF)

S2 Fig Gaussian resistance relationships. (PDF)

S3 Fig Sensitivity to simulation time frame. (PDF)

S1 Table Microsatellite locus information. (PDF)

S2 Table Geospatial data sources for landscape variables. (PDF)

S3 Table Alternative resistance curves for continuous landscape variables. (PDF)

S4 Table Alternative resistance ratios for categorical landscape variables. (PDF)
