# Supplementary material for: Simulating the spread of selection-driven genotypes using landscape resistance models for desert bighorn sheep
Source: PLoS One. 2017 May 2;12(5):e0176960. doi: 10.1371/journal.pone.0176960 (PMC5413035; doi:10.1371/journal.pone.0176960)
Supplement: S1 Appendix — (PDF) [file pone.0176960.s001.pdf]

S1 Appendix. Genetic laboratory methods.

Genetic samples used in this study were collected during multiple periods and analyzed in three genetics labs: 1) by C. Epps in the Roderick and Palsboll labs at the University of California, Berkeley (samples from 2000-2004; hereafter, "UCB lab"); 2) by the Epps lab at Oregon State University (samples from 2011-2014; hereafter, the "OSU lab"); 3) and by J. Wehausen at White Mountain Research Station in Bishop, CA (samples from 2003-2010; hereafter, the "WMRS lab"). The vast majority of samples consisted of bighorn sheep fecal pellets, but a small number of tissue and blood samples were obtained from live captures, hunter kills, or carcasses found in the field. Because labs used different primer sizes and allele-calling procedures, it was necessary to realign allele sizes for consistency. We accomplished this by genotyping a small subset of samples at each locus used by multiple laboratories and translating all allele sizes to match those used in the OSU laboratory.

We describe the genetic methods used by the OSU lab in detail below. Methods used by the UCB and WMRS labs are similar, and are described in Epps et al. (2005) and Jaeger and Wehausen (2012), respectively. S1 Table gives characteristics of each locus used in this study and the geographic regions and labs in which each locus was used.

DNA extraction:

We processed bighorn fecal pellets using the pellet-scraping method detailed in Wehausen et al. (2004) to collect 0.03 g of scrapings from the exterior surface of pellets. We extracted DNA from pellet scrapings using a modified AquaGenomic Stool and Soil protocol (MultiTarget Pharmaceuticals LLC, Colorado Springs, CO). Modifications included the addition of 450 µL of AquaGenomic solution to pellet scrapings, the use of 1.0 mm silica/zirconium beads (BioSpec Products Inc., Bartlesville, OK) for cell lysis, and the addition of 12 mAU proteinase K (Qiagen Inc., Valencia, CA) for recovery of mitochondrial DNA. Lastly, we added 150 µL of AquaPrecipi solution (MultiTarget Pharmaceuticals) to cell lysate to remove PCR inhibitors present in fecal samples. Tissue samples were extracted using the Qiagen DNeasy blood and tissue kits; we did not quantify DNA concentrations.

PCR recipe and cycling conditions:

Sixteen dinucleotide microsatellite markers were analyzed in three panels of 4-6 markers (S1 Table). Amplification of most loci was conducted in 10 µL reactions consisting of 5x Qiagen Multiplex PCR Master Mix, 10 µg of bovine serum albumen, 0.15-0.25 µM of each primer and 0.6 µL of genomic DNA. Reactions were brought to volume with nuclease-free water. Thermalcycling conditions for the multiplexed loci were as follows: initial denaturation of 15 minutes at 95 °C, followed by 35 cycles of [95 °C for 30 seconds, 60 °C for 90 seconds, 72 °C for 60 seconds], and a final elongation of 30 minutes at 60 °C. For each locus, one primer was fluorescently tagged on the 5' end with NED, PET, VIC (Applied Biosystems, Carlsbad, CA) or 6-FAM (Sigma-Aldrich, St. Louis, MO). Negative and positive controls were included on each genotyping run. PCRs were run on BioRad C1000 and MyCycler thermalcycler machines (Bio-Rad Laboratories Inc., Hercules, CA).

Two markers (BL4 and TGLA387) amplified weakly when pre-PCR multiplexed with other markers; these markers were each run in separate single-locus PCRs and then combined with the rest of the markers from that panel in a post-PCR multiplex. BL4 and TGLA387 were

amplified in 10μL reactions consisting of consisting of 1x magnesium-free PCR buffer, 3 mM $MgCl_2$, 160 μM of each dNTP, 10 μg bovine serum albumin, 0.35 μM of each primer, 0.7 units of Hot Start *Taq* polymerase (Apex Bioresearch Products) and 0.6μL of genomic DNA, and then brought to volume with nuclease-free water. Thermalcycling conditions were as follows: initial denaturation of 15 minutes at 95 °C, followed by 40 cycles of [95 °C for 30 seconds, 45 seconds at 60 °C (BL4) or 52 °C (TGLA387), and 72 °C for 30 seconds], with a final elongation step of five minutes at 72 °C.

Typing:

Each sample was amplified in three replicate PCRs for the six markers in panel 1 (S1 Table). We generated consensus genotypes across all three replicates: for a homozygous genotype to be considered verified, the allele had to be typed in three separate replicates. To confirm a heterozygous genotype, each allele had to be observed at least twice. Samples with incomplete or discrepant data were rerun in an additional 3-6 replicates. Any sample that consistently showed more than 2 alleles at a single locus was considered contaminated and removed.

Amplification products were visualized on a 2% agarose gel prestained with GelRed (Biotium Inc., Hayward, CA). Products were diluted accordingly, ethanol-precipitated to remove salts, and submitted for fragment size analysis on the ABI DNA 3730 DNA analyzer (Applied Biosystems) at the Oregon State University Center for Genome Research and Biocomputing (Corvallis, OR). We used GeneScan 500 LIZ dye size standard (Applied Biosystems), and called allele sizes in GeneMapper v.4.1 (Applied Biosystems).

Identifying duplicates:

We grouped samples into major regions comprising all populations within or near each national park unit (e.g., Death Valley, Glen Canyon, Grand Canyon, etc.) before identifying duplicates. In some cases, major genetic divisions with different allele frequencies existed within a region (e.g., populations on either side of the Colorado River in Grand Canyon), so we analyzed these subregions independently. We used program CERVUS version 3.0.3 (Kalinowski et al. 2007) to calculate the allele frequencies and probability of identity (P_{ID}) for each region (or subregion) using the six markers in Panel 1. Since missing data most frequently occurred for locus TGLA387 in any sample, we recalculated the P_{ID} using only the other five markers in Panel 1. We then searched for duplicate individuals within a region, using the minimum number of loci required to have a P_{ID} for unrelated individuals of <0.01 (because most regions were generally estimated to have <100 sampled individuals), and a P_{ID} for siblings of <0.05. We ran additional searches for duplicates using decreased stringency (i.e., allowed fuzzy matching) until CERVUS began returning matches that were unlikely due to sampling location (e.g., putative duplicates sampled hundreds of kilometers apart) or the inability to explain mismatches with allelic dropout (e.g., heterozygotes with different alleles). When duplicate samples were discovered, we removed all but one from further analyses. Samples that had too much missing data were retained in the data set and run with additional markers until we could verify whether or not they were unique.

We then ran putative unique genotypes for the remaining two panels (10 loci) and reran the CERVUS analyses using all 16 markers to recalculate P_{ID} and the minimum number of loci to identify matches. In this manner, we removed additional duplicates that did not amplify at enough markers in Panel 1 to be excluded. Finally, samples with fewer than 5 loci successfully

typed were removed from the data set. The mean number of loci successfully typed per sample in the final dataset was 15.4, with at least 13 loci successfully typed for 95 percent of samples.

Error rates and equilibrium tests:

We used GIMLET version 1.3.3 (Valière 2002) to estimate genotyping error rates (both false allele occurrence rate and allelic dropout rate) for a subset of regions with varying sample sizes (Glen Canyon, Death Valley, Capitol Reef, Utah BLM lands). False allele occurrence rate was zero for all regions tested, and allelic dropout rate averaged 4.1 percent across loci and regions.

We used GENEPOP version 4.2 (Raymond and Rousset 1995) to test for deviations from linkage equilibrium (LE) and Hardy-Weinberg equilibrium (HWE) in each sampled population within each region and corrected for multiple comparisons. We observed deviations from HWE or LE in a number of populations; however, no locus (for HWE) or pair of loci (for LE) was consistently out of equilibrium across populations, suggesting that these deviations most likely resulted from population substructure rather than non-neutral loci or non-independent loci. We therefore retained all loci in subsequent analyses.

References (including those in S1 Table):

Buchanan, F.C., and A.M. Crawford. 1992a. Ovine dinucleotide repeat polymorphism at the MAF209 locus. Animal Genetics 23:183.

Buchanan, F.C., and A.M. Crawford. 1992b. Ovine dinucleotide repeat polymorphism at the MAF33 locus. Animal Genetics 23:186.

Buchanan, F.C., and A.M. Crawford. 1993. Ovine microsatellites at the OarFCB11, OarFCB128, OarFCB193, OarFCB266, and OarFCB304 loci. Animal Genetics 24:145.

Buchanan, F.C., P.A. Swarbrick, and A.M. Crawford. 1991. Ovine dinucleotide repeat polymorphism at the MAF65 locus. Animal Genetics 23:85.

Buchanan, F.C., P.A. Swarbrick, and A.M. Crawford. 1992. Ovine dinucleotide repeat polymorphism at the MAF48 locus. Animal Genetics 22:379-380.

Crawford, A.M., K.G. Dodds, A.J. Ede, C.A. Pierson, G.W. Montgomery, H.G. Garmonsway, A.E. Beattie, K. Davies, J.F. Maddox, S.W. Kappes, R.T. Stone, T.C. Nguyen, J.M. Penty, E.A. Lord, J.E. Broom, J. Buitkamp, W. Schwaiger, J.T. Epplen, P. Matthew, M.E. Matthews, D.J. Hulme, K.J. Beh, R.A. McGraw, and C.W. Beattie. 1995. An autosomal genetic linkage map of the sheep genome. Genetics 140:703-724.

Ede, A.J., C.A. Peirson, H. Henry, and A.M. Crawford. 1994. Ovine microsatellites at the OarAE64, OarHH22, OarHH56, OarHH62, and OarVH4 loci. Animal Genetics 25:51-52.

Ede, A.J., C.A. Pierson, and A.M. Crawford. 1995. Ovine microsatellites at the OarCP9, OarCP16, OarCP20, OarCP21, OarCP23, and OarCP26 loci. Animal Genetics 26:129-130.

Epps, C.W., P.J. Palsbøll, J.D. Wehausen, G.K. Roderick, R.R. Ramey, and D.R. McCullough. 2005. Highways block gene flow and cause a rapid decline in genetic diversity of desert bighorn sheep. Ecology Letters 8:1029-1038.

Georges, M., and J. Massey. 1992. Polymorphic DNA markers in Bovidae. In: WO Publication No. 92/13120. World Intellectual Property Organisation, Geneva.

Henry, H.M., J.M Penty, C.A. Pierson, and A.M. Crawford. 1993. Ovine microsatellites at the OarHH35, OarHH41, OarHH44, OarHH47 and OarHH64 loci. Animal Genetics 24:222.

Jaeger, J.R. and J.D. Wehausen. 2012. Development of a habitat management plan to maintain viability of the desert bighorn sheep population in the River Mountains, Nevada: analysis of mitochondrial DNA diversity and connectivity. Final Report to the National Park Service, Lake Mead National Recreation Area. University of Nevada, Las Vegas.

Kalinowski, S.T., M.L. Taper, and T.C. Marshall. 2007. Revising how the computer program cervus accommodates genotyping error increases success in paternity assignment. Molecular Ecology 16:1099-1106.

Pentry, J.M., H.M. Henry, A.J. Ede, and A.M. Crawford. 1993. Ovine microsatellites at the OarAE16, OarAE54, OarAE57, OarAE119, and OarAE129 loci. Animal Genetics 24:219.

Raymond, M. and F. Rousset. 1995. GENEPOP (version 1.2): population genetics software for exact tests and ecumenicism. Journal of Heredity 86:248-249.

Smith, T.P., N. Lopez-Corrales, M.D. Grosz, C.W. Beattie, and S.M. Kappes. 1997. Anchoring of bovine chromosomes 4, 6, 7, 10, and 14 linkage group telomeric ends via FISH analysis of lambda clones. Mammalian Genome 8:333-336.

Steffen, P., A. Eggen, G. Stranzinger, R. Fries, A. Dietz, A. and J. Womack. 1993. Isolation and mapping of polymorphic microsatellites in cattle. Animal Genetics 24:121-124.

Swarbrick, P., F. Buchanan, and A. Crawford. 1991. Ovine dinucleotide repeat polymorphism at the MAF35 locus. Animal Genetics 22:369-370.

Valière, N. 2002. GIMLET: a computer program for analysing genetic individual identification data. Molecular Ecology Notes 2:377-379.

Wehausen, J.D., R.R. Ramey, and C.W. Epps. 2004. Experiments in DNA extraction and PCR amplification from bighorn sheep feces: the importance of DNA extraction method. Journal of Heredity 95:503-509.
