# Supplementary material for: Simulating the spread of selection-driven genotypes using landscape resistance models for desert bighorn sheep
Source: PLoS One. 2017 May 2;12(5):e0176960. doi: 10.1371/journal.pone.0176960 (PMC5413035; doi:10.1371/journal.pone.0176960)
Supplement: S2 Appendix — (PDF) [file pone.0176960.s002.pdf]

S2 Appendix. Resistance model equations.

We used three equations to describe the relationships between continuous landscape variables and resistance, where resistance varies between 1 and a user-defined maximum resistance value. Equation S1 describes possible resistance curves when resistance values and landscape variable values are expected to be positively related:

$$r = \frac{x^{\alpha}}{x_{max}^{\alpha}} * (r_{max} - 1) + 1 \qquad \text{(Eqn. S1)}$$

where r is resistance, r_{max} is the maximum resistance value, x is the value of the landscape variable, x_{max} is the maximum landscape variable value observed within the three study regions, and α is an exponent that controls the shape of the relationship. For landscape variables expected to be negatively related to resistance, we used Eqn. S2:

$$r = r_{max} - \frac{x^{\alpha}}{x_{max}^{\alpha}} * (r_{max} - 1) \qquad \text{(Eqn. S2)}$$

Depending on the value of α, these equations specify relationships that can be either concave-up or concave-down (S1 Fig). When $\alpha = 1$, the relationships are linear.

Some landscape variables could exhibit lowest resistance at an intermediate value; for instance, very shallow slopes may expose bighorn sheep to predation and very steep slopes may be difficult to negotiate, while intermediate slopes might offer the least resistance. Following Castillo et al. (2014), we modeled these relations using an inverse Gaussian function:

$$r = r_{max} - (r_{max} - 1) * e^{\left(-\frac{\left(x - x_{opt}\right)^2}{2x_{sd}^2}\right)} \qquad \text{(Eqn. S3)}$$

where r_{max} is the maximum resistance value, x is the value of the landscape variable, x_{opt} is the optimal (i.e., lowest resistance) value of the landscape variable, and x_{sd} is the standard deviation of the normal curve (S2 Fig).
