# Supplementary material for: Simulating the spread of selection-driven genotypes using landscape resistance models for desert bighorn sheep
Source: PLoS One. 2017 May 2;12(5):e0176960. doi: 10.1371/journal.pone.0176960 (PMC5413035; doi:10.1371/journal.pone.0176960)
Supplement: S1 Fig — (PDF) [file pone.0176960.s006.pdf]

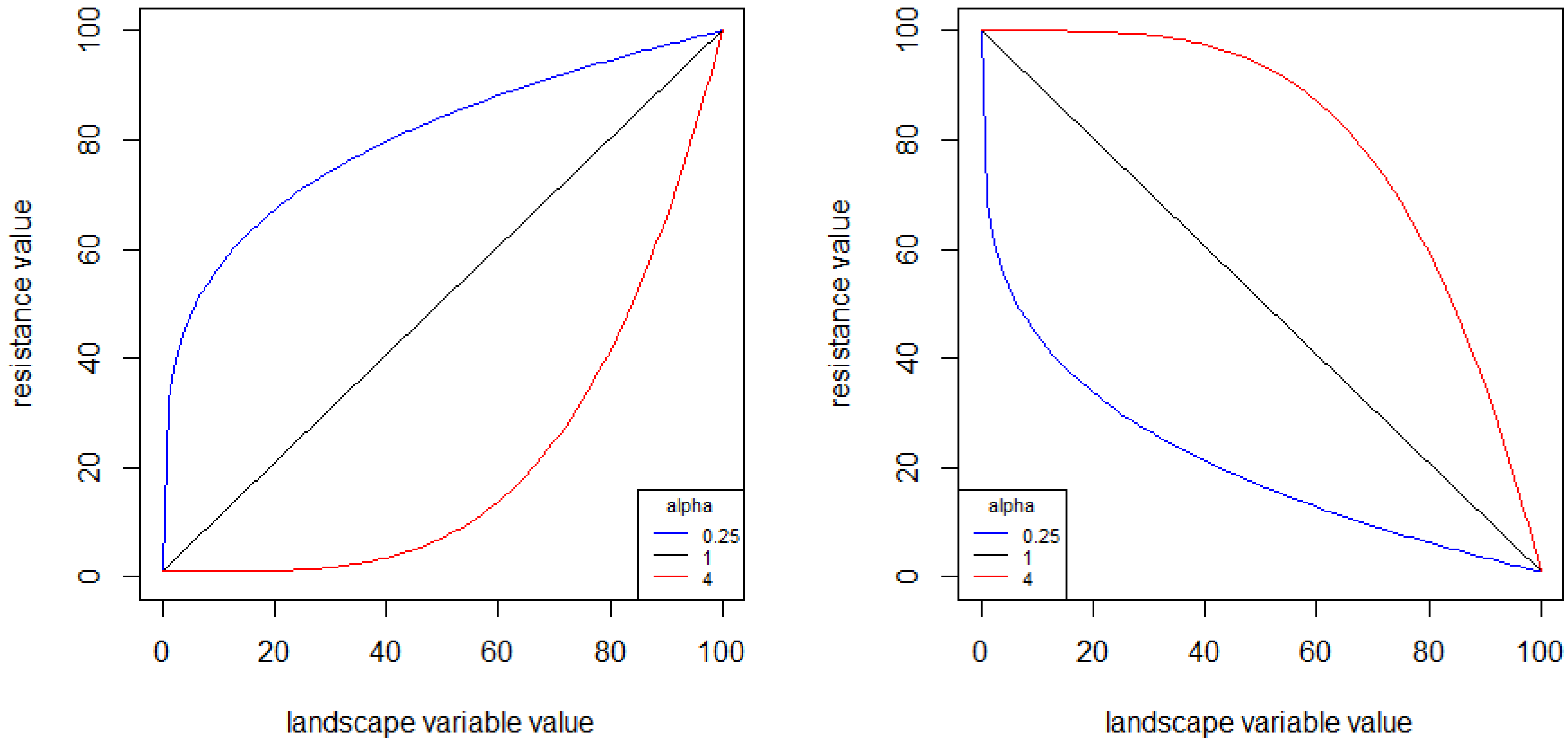

S1 Fig. Monotonic resistance relationships. Curves resulting from Eqn. S1 (left panel) and Eqn. S2 (right panel) for a range of α values and a hypothetical landscape variable with x_{max} = 100 and r_{max} = 100.
