# Supplementary material for: Simulating the spread of selection-driven genotypes using landscape resistance models for desert bighorn sheep
Source: PLoS One. 2017 May 2;12(5):e0176960. doi: 10.1371/journal.pone.0176960 (PMC5413035; doi:10.1371/journal.pone.0176960)
Supplement: S2 Fig — (PDF) [file pone.0176960.s007.pdf]

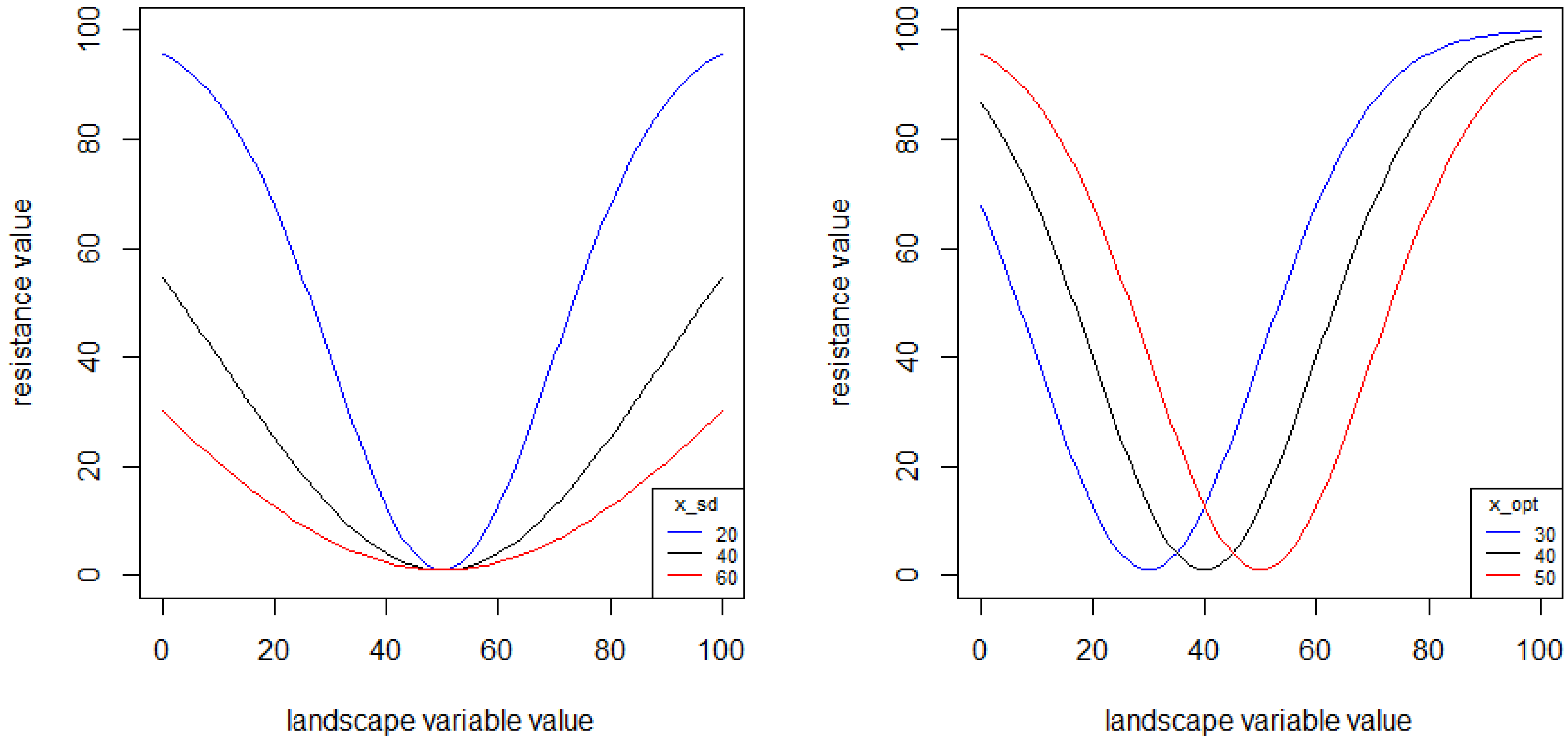

S2 Fig. Gaussian resistance relationships. Curves resulting from Eqn. S3 for a range of x_{sd} values (left panel) or x_{opt} values (right panel) and a hypothetical landscape variable with x_{max} = 100 and r_{max} = 100.
