# Supplementary material for: Simulating the spread of selection-driven genotypes using landscape resistance models for desert bighorn sheep
Source: PLoS One. 2017 May 2;12(5):e0176960. doi: 10.1371/journal.pone.0176960 (PMC5413035; doi:10.1371/journal.pone.0176960)
Supplement: S3 Fig — (PDF) [file pone.0176960.s008.pdf]

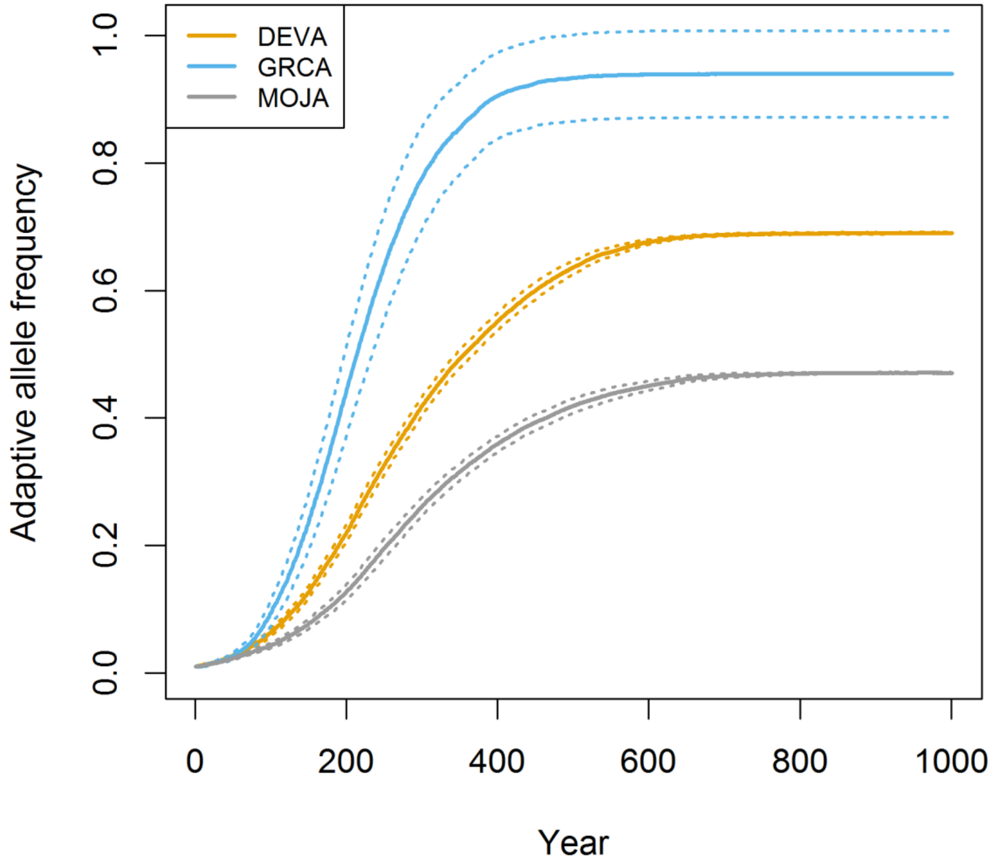

S3 Fig. Sensitivity to simulation time frame. We chose to run CDPOP simulations for 100 years in order to observe changes in adaptive allele frequency over a time frame that remains relevant for the conservation and management of bighorn sheep. We also had practical reasons for limiting simulations to 100 years: computation time and computer memory needed to run simulations over long periods (e.g., thousands of years) were prohibitive. However, longer time frames may provide a different perspective on the effects of landscape structure on the spread of adaptive variation. As a brief exploration of the potential for different results over longer time frames, we reran simulations for a one combination of dispersal threshold, selection strength, and scenario (high dispersal, strong selection, novel allele) for 1,000 years to compare with the results of our 100-year output. Results of this reanalysis for all three regions are shown above, with solid and dashed lines representing means and 95 percent confidence limits, respectively, from 50 MC replicates per region. These results suggest that the patterns that begin emerging by year 100 continue to manifest over longer time periods. The shorter simulations appear to capture the differences in trends that lead toward different asymptotic allele frequencies.
