# Supplementary material for: Simulating the spread of selection-driven genotypes using landscape resistance models for desert bighorn sheep
Source: PLoS One. 2017 May 2;12(5):e0176960. doi: 10.1371/journal.pone.0176960 (PMC5413035; doi:10.1371/journal.pone.0176960)
Supplement: S1 Table — (PDF) [file pone.0176960.s009.pdf]

S1 Table. Microsatellite locus information. Genetic diversity measures shown for each region/locus include number of alleles observed (N_a), observed heterozygosity (H_o), and expected heterozygosity (H_e). Grayed-out boxes indicate that samples from that region were not genotyped at that locus.

			Death Valley			Grand Canyon			Southern Mojave		
Locus	Reference[a]	Labs[b]	N_a	H_o	H_e	N_a	H_o	H_e	N_a	H_o	H_e
AE129	Pentry et al. 1993	OSU, WMRS	9	0.79	0.82	9	0.74	0.76			
AE16	Pentry et al. 1993	OSU, WMRS, UCB	6	0.77	0.77	7	0.60	0.69	6	0.68	0.74
BL4	Smith et al. 1997	OSU, WMRS	4	0.60	0.68	4	0.20	0.39			
CP20	Ede et al. 1995	UCB							10	0.71	0.80
D5S2	Steffen et al. 1993	UCB							9	0.71	0.85
FCB11	Buchanan and Crawford 1993	OSU, WMRS, UCB	4	0.59	0.64	3	0.61	0.65	4	0.51	0.60
FCB128	Buchanan and Crawford 1993	UCB, WMRS							2	0.28	0.30
FCB193	Buchanan and Crawford 1993	OSU, WMRS	8	0.69	0.70	6	0.70	0.78			
FCB266	Buchanan and Crawford 1993	OSU, WMRS, UCB	7	0.53	0.52	5	0.49	0.50	5	0.47	0.53
FCB304	Buchanan and Crawford 1993	OSU, WMRS, UCB	4	0.73	0.71	4	0.48	0.58	5	0.48	0.55
HH47	Henry et al. 1993	UCB, WMRS							10	0.70	0.84
HH62	Ede et al. 1994	OSU, WMRS, UCB	10	0.67	0.86	15	0.80	0.85	12	0.72	0.85
JMP29	Crawford et al. 1995	OSU, WMRS	12	0.60	0.69	9	0.64	0.63			
MAF209	Buchanan and Crawford 1992a	OSU, WMRS, UCB	7	0.69	0.80	6	0.66	0.75	8	0.58	0.71
MAF33	Buchanan and Crawford 1992b	OSU, WMRS, UCB	6	0.67	0.67	6	0.68	0.70	4	0.54	0.65
MAF36	Swarbrick et al. 1991	OSU, WMRS, UCB	6	0.66	0.68	6	0.67	0.76	7	0.51	0.67
MAF48	Buchanan et al. 1992	OSU, WMRS, UCB	6	0.67	0.71	4	0.63	0.67	5	0.67	0.76
MAF65	Buchanan et al. 1991	OSU, WMRS, UCB	10	0.70	0.78	7	0.75	0.81	9	0.62	0.70
TCRBV62	Crawford et al. 1995	OSU, WMRS	7	0.73	0.72	7	0.76	0.83			
TGLA387	Georges and Massey 1992	OSU				6	0.70	0.79			

[a] Full citations are listed at end of S1 Appendix.
[b] Labs used different subsets of the 20 loci in this study. OSU = Oregon State University; UCB = University of California, Berkeley; WMRS = White Mountain Research Station.
