# Supplementary material for: Simulating the spread of selection-driven genotypes using landscape resistance models for desert bighorn sheep
Source: PLoS One. 2017 May 2;12(5):e0176960. doi: 10.1371/journal.pone.0176960 (PMC5413035; doi:10.1371/journal.pone.0176960)
Supplement: S2 Table — (PDF) [file pone.0176960.s010.pdf]

S2 Table. Geospatial data sources for landscape variables.

Variable	Data description	Original spatial resolution	Source
Slope	Slope in degrees, derived from digital elevation model using ArcGIS 10.1	1/3 arcsecond (~10 m)	DEM from National Elevation Dataset (http://ned.usgs.gov/)
NDVI	Average of annual total integrated NDVI values during 2000-2013	250 m	USGS Eros Center (http://phenology.cr.usgs.gov/)
Distance to water	Distance to the nearest permanent surface water source	Vector data	National Hydrography Dataset (http://nhd.usgs.gov/); National Park Service
Water barriers	Water features believed to act as strong barriers to bighorn sheep	Vector data	National Hydrography Dataset
Anthropogenic development	Converted (versus natural) cover types	30 m	2011 National Land Cover Database (http://www.mrlc.gov/nlcd2011.php)
Major roads	Divided, fenced highways	Vector data	U.S. Census (www.census.gov)
Forested areas	Evergreen, deciduous, and mixed forest cover types	100 m	2011 National Land Cover Database
