# Supplementary material for: Simulating the spread of selection-driven genotypes using landscape resistance models for desert bighorn sheep
Source: PLoS One. 2017 May 2;12(5):e0176960. doi: 10.1371/journal.pone.0176960 (PMC5413035; doi:10.1371/journal.pone.0176960)
Supplement: S3 Table — (PDF) [file pone.0176960.s011.pdf]

S3 Table. Alternative resistance curves for continuous landscape variables. All possible combinations of parameter values for each variable were used as candidate univariate resistance surfaces in all three regions, with two exceptions: 1) major roads resistance surfaces were not tested for DEVA or GRCA because no major roads exist within these regions; and 2) water barrier resistance features were not tested within DEVA because no water barriers exist within the DEVA region.

Variable	Curve description	Parameters
Slope	Linear	R_{max} = 10; 50; 100 | α = 1
	Concave up	R_{max} = 10; 50; 100 | α = 0.25
	Concave down	R_{max} = 10; 50; 100 | α = 4
	Gaussian	R_{max} = 10; 50; 100 | x_{opt} = 30; 40; 50 | x_{sd} = 20; 40; 60
	Break point	ratio = 5; 10; 20 | lower break = 10; 15; 20 | upper break = 45; 55; 65
NDVI	Linear	R_{max} = 10; 50; 100 | α = 1
	Concave up (weak)	R_{max} = 10; 50; 100 | α = 0.25
	Concave down (strong)	R_{max} = 10; 50; 100 | α = 4
Distance to water	Linear	R_{max} = 10; 50; 100 | α = 1
	Concave up (weak)	R_{max} = 10; 50; 100 | α = 4
	Concave down (strong)	R_{max} = 10; 50; 100 | α = 0.25
