# Supplementary material for: Simulating the spread of selection-driven genotypes using landscape resistance models for desert bighorn sheep
Source: PLoS One. 2017 May 2;12(5):e0176960. doi: 10.1371/journal.pone.0176960 (PMC5413035; doi:10.1371/journal.pone.0176960)
Supplement: S4 Table — (PDF) [file pone.0176960.s012.pdf]

S4 Table. Alternative resistance ratios for categorical landscape variables.

Variable	Resistance ratio												
	**5**	**10**	**25**	**50**	**100**	**250**	**500**	**1,000**	**5,000**	**10,000**	**50,000**	**100,000**	**1,000,000**
Anthropogenic development	✓	✓	✓	✓	✓	✓	✓	✓	✓	✓			
Major roads		✓		✓	✓		✓	✓	✓	✓	✓	✓	
Forested areas	✓	✓	✓	✓	✓	✓	✓	✓					
Water barriers		✓		✓	✓		✓	✓	✓	✓	✓	✓	✓
